# Identification of key genes and pathways revealing the central regulatory mechanism of brain-derived glucagon-like peptide-1 on obesity using bioinformatics analysis

**DOI:** 10.3389/fnins.2022.931161

**Published:** 2022-08-05

**Authors:** Yuwei Shao, Jun Tian, Yanan Yang, Yan Hu, Ye Zhu, Qing Shu

**Affiliations:** ^1^Department of Rehabilitation, Zhongnan Hospital of Wuhan University, Wuhan, China; ^2^Department of Traditional Chinese Medicine, China Resources Wugang General Hospital, Wuhan, China; ^3^College of Health Sciences, Wuhan Sports University, Wuhan, China

**Keywords:** glucagon-like peptide-1 (GLP-1), appetite, obesity, bioinformatics, chemical genetics

## Abstract

**Objective:**

Central glucagon-like peptide-1 (GLP-1) is a target in treating obesity due to its effect on suppressing appetite, but the possible downstream key genes that GLP-1 regulated have not been studied in depth. This study intends to screen out the downstream feeding regulation genes of central GLP-1 neurons through bioinformatics analysis and verify them by chemical genetics, which may provide insights for future research.

**Materials and methods:**

GSE135862 genetic expression profiles were extracted from the Gene Expression Omnibus (GEO) database. The gene ontology (GO) and Kyoto Encyclopedia of Genes and Genomes pathway (KEGG) enrichment analyses were carried out. STRING database and Cytoscape software were used to map the protein-protein interaction (PPI) network of the differentially expressed genes (DEGs). After bioinformatics analysis, we applied chemogenetic methods to modulate the activities of GLP-1 neurons in the nucleus tractus solitarius (NTS) and observed the alterations of screened differential genes and their protein expressions in the hypothalamus under different excitatory conditions of GLP-1 neurons.

**Results:**

A total of 49 DEGs were discovered, including 38 downregulated genes and 11 upregulated genes. The two genes with the highest expression scores were *biglycan* (*Bgn*) and *mitogen-activated protein kinase activated protein kinase 3* (*Mapkapk3*). The results of GO analysis showed that there were 10 molecular functions of differential genes. Differential genes were mainly localized in seven regions around the cells, and enriched in 10 biology processes. The results of the KEGG signaling pathway enrichment analysis showed that differential genes played an important role in seven pathways. The top 15 genes selected according to the Cytoscape software included *Bgn* and *Mapkapk3*. Chemogenetic activation of GLP-1 in NTS induced a decrease in food intake and body mass, while chemogenetic inhibition induced the opposite effect. The gene and protein expression of GLP-1 were upregulated in NTS when activated by chemogenetics. In addition, the expression of Bgn was upregulated and that of Mapkapk3 was downregulated in the hypothalamus.

**Conclusion:**

Our data showed that GLP-1 could modulate the protein expression of Bgn and Mapkapk3. Our findings elucidated the regulatory network in GLP-1 to obesity and might provide a novel diagnostic and therapeutic target for obesity.

## Introduction

Over the past 40 years, the prevalence of obesity has increased substantially worldwide. The prevalence in children increased from less than 1% in 1975 to 6 to 8% in 2016, in adult males increased from 3 to 11%, and in adult females increased from 6 to 15% during the same period (Jaacks et al., 2019). China has the highest number of obese people in the world, with about 46% of adults and 15% of children suffering from obesity or being overweight (Wang et al., 2019). As one of the most important risk factors for diabetes, cardiovascular disease, cancer, osteoarthritis, movement disorders, and sleep apnea, obesity seriously affects human health (Seidell and Halberstadt, 2015). One of the most effective solutions to obesity is to control appetite and reduce caloric intake (Bray and Siri-Tarino, 2016). Various therapies have shown efficacy to treat obesity, including pharmacotherapy (Narayanaswami and Dwoskin, 2017), operation therapy (Nudel and Sanchez, 2019), and even traditional Chinese medicine (TCM) (Santos et al., 2020; Ni et al., 2021).

Appetite is regulated by a complex system of central and peripheral signals that interact to modulate an individual’s response to nutrition. Peripheral regulation includes satiety signaling and adiposity signaling, whereas central regulation is accomplished by a variety of factors, including the neuropeptidergic, monoaminergic, and endocannabinoid systems. Satiety signals mainly include cholecystokinin (CCK), GLP-1, and casein tyrosine (Valassi et al., 2008).

Glucagon-like peptide 1 is an intestinal hormone that is released in response to food intake. It can enhance the stimulation of insulin synthesis and secretion by glucose while inhibiting the secretion of glucagon and delaying gastric emptying (D’Alessio, 2016). GLP-1 exerts an inhibitory effect on food intake *via* its receptor GLP-1R, which is widely distributed in the brain, gastrointestinal tract, and pancreas (Fakhry et al., 2017). In clinical practice, medication and surgery are widely used. The mechanisms of these interventions in treating obesity are all related to the regulation of GLP-1 (Albaugh et al., 2019; Luo et al., 2020). Some non-pharmaceutical TCM therapies, such as acupuncture, have also shown the potential to affect metabolism by modulating GLP-1 (Firouzjaei et al., 2016). GLP-1 is well-known to medical workers as an important hormone in appetite regulatory pathways, but little is known about how they act in the central nervous system and peripheral nervous system to produce enhanced satiety and inhibit appetite mechanisms. At present, high-throughput gene chip technology and bioinformatics analysis have been widely used in the pathogenesis of diseases, molecular diagnosis, and other aspects (Huang et al., 2018). New scientific and technological methods represented by genomics approaches provide a large amount of research data for mechanistic studies of appetite regulation. Therefore, in this study, we used high-throughput sequencing data from public platforms to decipher the central regulatory mechanism of GLP-1R agonist (GLP1-RA) administration through a bioinformatics approach. Overall, this study advances our understanding of the central mechanisms that synergize the inhibitory effects of appetite and body weight to treat obesity and provides a basis for further research.

## Materials and methods

### Gene chip data acquisition

The high-throughput dataset GSE135862 related to the mechanisms of appetite regulation by GLP-1/CCK was retrieved and downloaded from GEO, a comprehensive database of gene expression. The chip contains a total of 48 samples. All of the studies were conducted in 9- to 10-week-old C57/Bl6 male mice, which were maintained on standard chow and fasted for 6 h before treatment administration. Multiple variables such as injection of drugs, injection sites, and group settings were included in the experimental conditions. Mice were randomized into treatment groups and received an IP injection of saline, AC710222, exenatide, AC3174, AC170222, or a combination of these drugs twice daily for 5 days. The injection sites were the dorsal vagal complex (DVC) and the medial basal hypothalamus (MBH), respectively. The experimental content is RIP-Seq (RNA co-immunoprecipitation combined with high-throughput sequencing). The purpose of this study was to investigate the pathway of GLP-1 in the brain. In this study, we selected the groups injected with GLP1-RA AC3174 and the blank control group for data comparison and analysis.

### Screening of differentially expressed genes

Samples injected with drug as saline were set as the control group and samples injected with drug as GLP1-RA were set as the intervention group using *P* < 0.05, log value of gene fold change (logFC) <−1, or logFC > 1 as the criterion (Zhang et al., 2019). The GSE135862 dataset was analyzed by the R language DESeq2 package to screen differentially expressed genes between different groups of the dataset and find out differentially expressed genes that may be involved in the regulation of appetite by GLP-1R.

### Gene ontology and Kyoto encyclopedia of genes and genomes pathway enrichment analysis of differentially expressed genes

Gene ontology is a commonly used analytical method whose main function is to annotate genes or their products and identify the characteristic biological characteristics of high-throughput genomic or transcriptome data. GO annotates and classifies genes according to Biological Process (BP), Molecular Function (MF), and Cellular Component (CC). The KEGG database is available for querying pathway information and signaling pathway retrieval, and so on. The main goal of the KEGG database is to endow genes and genomes with functional significance at the molecular level and higher levels, which establish links between genes in the genome and advanced functions of cells and organisms (Kanehisa et al., 2016, 2017). This study used the DAVID (version: 6.8)^1^ database for integrative analysis. Using Fisher Exact or EASE Score statistical methods, GO items were screened with *P* < 0.05, false discovery rate (FDR) <1, and KEGG items with *P* < 0.05 (Sherman et al., 2007).

### Analysis of differentially expressed gene protein interaction network

STRING (version: 11.5)^2^ is an online analysis tool that can be used to present and evaluate interactions between proteins (Szklarczyk et al., 2019). The magnitude of the likelihood of a protein is evaluated by scoring its mutual-action network. In this study, the analyzed differential genes were input into the STRING analysis tool to find potential links between them. The screening condition for constructing the interaction network of differentially gene-encoded proteins was the threshold of interaction likelihood. The STRING database export results were then imported into Cytoscape 3.9.1 software for visual analysis. The cytoHubba plugin in the software was used to find out the top 15 hub genes scored according to the Maximal Clique Centrality (MCC) and Degree scoring methods, respectively (Hu and Chan, 2013).

### Preparation of recombinant adeno-associated virus

Promoter sequences of GLP-1 protein were determined by searching the gene database and published relevant literature (Rasouli et al., 2011; Shi et al., 2017). Customized recombinant adeno-associated virus (rAAV), which included GLP-1 promoter and cyclization recombination enzyme (cre) applied in this experiment, was provided by Wuhan BrainVTA Technology Co., Ltd. The sequences of the other three rAAVs are as follows: rAAV-Efla-DIO-EGFP-WPRE-pA (rAAV-GFP), rAAV-hSyn-DIO-hM3D (Gq) -mCherry-WPRE-pA (rAAV-HM3D), and rAAV-hSyn-DIO-hM4D (Gi) -mCherry-WPRE-pA (rAAV-HM4D), which were purchased from commercial companies (BrainVTA Co., Ltd, Wuhan, China).

### Animals and intervention

We acquired 15 male Sprague–Dawley rats, aged 6 weeks, and weighing 180 to 200 g from Beijing Vital River Laboratory Animal Technology Co., Ltd. (Beijing, China, No. 11400700298971). All rats were placed in a controlled environment at 22 ± 2°C and 50 ± 10% relative humidity with a 12-h light/12-h dark cycle in the Experimental Animal Center, Zhongnan Hospital of Wuhan University, with standard food and water provided *ad libitum* for the duration of the study. The study protocol was authorized by the Institutional Animal Care and Use Committee of Wuhan University, Wuhan City, Hubei Province, China (AUP Number: WP2020-08085).

**FIGURE 1:**
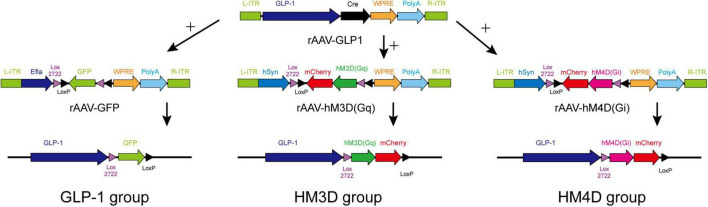
Gene sequence and combinatorial strategy of rAAVs.

After 1 week of adaptive feeding, all rats were numbered according to body weight, and 15 rats were divided into the GLP-1 group (*n* = 5), HM3D group (*n* = 5), and HM4D group (*n* = 5) by stratified randomization. Stereotactic injection of NTS was performed according to validated parameters (AP: Lambda-3.2 mm, ML: ± 0.5–0.7 mm, DV: 9.6 mm, and oblique angle backward 24°) for injection (Shu et al., 2020). Rats in each group were injected with rAAV as follows: rats in the GLP-1 group were injected with rAAV-GLP-1 and rAAV-GFP into NTS with 260 nl of each rAAV; rats in the HM3D group were injected with rAAV-GLP-1 and rAAV-3D into NTS, 260 nl of each rAAV; and rats in HM4D group were injected with rAAV-GLP-1 and rAAV-4D, 260 nl of each rAAV. The chronological order of injection was based on the body weight of the rats, and the rats whose body weight first reached about 400 g were injected first, and finally, the NTS virus injection was completed in all rats at about 5 days. All rats were observed for 1 week after the completion of the injection, with the supplement of food and water *ad libitum* during the observation period. Specific rAAV gene sequence and combinatorial strategy were shown in Figure 1. Three weeks after rAAV injection in all rats, the exogenous gene carried by rAAV was expressed in target neurons. We collected the baseline data of all rats (body mass, lee’s index, and 24 h food intake) 3 weeks after the injection of the last rat, and assigned this day as day 0. Rats in all three groups received four times intraperitoneal injections of Clozapine-N-oxide (CNO) at a dose of 1 mg/kg on the 1st, 3rd, 6th, and 9th day of the experiment (Gomez et al., 2017).

### Behavior test

After collecting the baseline data on day 0 of the experiment, we intraperitoneally injected CNO into all rats on the 1st day and recorded the cumulative food intake at 0.5, 1, 2, and 24 h after injection to evaluate the short-term feeding behavior of rats after regulating neuronal excitability. Subsequently, we recorded the cumulative 24 h food intake after the rats received CNO injection and body weight on the 3rd, 6th, and 9th days of the experiment, which aims to observe the long-term feeding behavior and body weight changes of rats after multiple regulations of neuronal excitability.

### Western blotting

All rats were anesthetized and sacrificed by cervical dislocation after 9 days of treatment. Thirty minutes before sacrifice, rats in HM3D and HM4D groups were in intraperitoneal injection with CNO to activate or inhibit GLP-1 neurons. We froze the tissue of NTS and hypothalamus in liquid nitrogen after stripping them from the rat brains and stored them at −80°C. NTS tissue was detected with GLP-1 antibody, while hypothalamus tissue was detected with Bgn antibody and Mapkapk3 antibody applying a standard WB protocol. The primary antibody concentrations were as follows: GLP-1 (1:1000, AF0166, Affinity, Japan), Bgn (1:1000, 16409-1-AP, Proteintech, China), and Mapkapk3 (1:1000, 15424-1-AP, China). We used the housekeeping protein β-actin (1:1000; Proteintech, China) for normalization. We performed WB in triplicate for all target proteins. Protein expression was calculated based on the target protein and β-actin ratios of optical density, which we analyzed using the BandScan software.

### Reverse transcription-quantitative polymerase chain reaction

We isolated total RNA of rat NTS and hypothalamus stored at −80°C with an AZfresh total RNA extraction kit (15596-026, Ambion, United States) and determined RNA concentrations at an absorbance ratio of 260/280 nm. We then reverse transcribed an aliquot (1 μg) of extracted RNA into first-strain complementary DNA (cDNA) using a ReverTra Ace qPCR RT kit (R223-01, VAZYME). We quantified gene expression of GLP-1 of NTS, Bgn, and Mapkapk3 of the hypothalamus by using an SYBR Green real-time PCR Master Mix Plus (Q111-02; VAZYME) and standard protocol. Measurements were conducted in triplicate under standard reaction conditions, and normalization was ensured to β-actin. We obtained primaries from the Biofavor Technology Company (Wuhan, China). All temperature circulation and gene amplification were processed in a CFX96 Touch real-time PCR detection system (Bio-Rad). All RT-PCR assays and primer sequences are shown in Table 1.

**TABLE 1:** Real-time PCR primer sequences.

Gene	Primer	Sequence (5′-3′)	PCR Products
β-actin	Forward	CACGATGGAGGGGCCGGACTCATC	241 bp
	Reverse	TAAAGACCTCTATGCCAACACAGT	
Rat GLP-1	Forward	TCGTGGCTGGATTGTTT	142 bp
	Reverse	TGGCGTTTGTCTTCGTT	
Bgn	Forward	GAGAACAGTGGCTTTGAACC	178 bp
	Reverse	GCTTGGAGTAGCGAAGTAGAT	
Mapkapk3	Forward	GTGCTGGGTCTGGGTGTGA	350 bp
	Reverse	CGGTGGGCAATGTTCTGG	

### Immunofluorescence staining

Rats were anesthetized with isoflurane and received transcardial perfusion with saline and 5% paraformaldehyde. Rat brains were immersion in 5% paraformaldehyde for 72 h for perfusion fixation followed by dehydration in 25% sucrose solution. The coronal plane where the NTS and hypothalamus are located was selected for the frozen section. The frozen sections in the NTS area with a thickness of 10 μm were selected. After thawing by natural gradient, 10% donkey serum or goat serum was used for blocking. After repairing with sodium citrate antigen retrieval solution, which leads the virus fluorescence to be destroyed, the three groups of brain slices were incubated with c-fos primary antibody. In GLP-1 group, GLP-1 neurons were labeled with anti-GFP (1:2000, ab5450, Abcam, United States) + green fluorescent secondary antibody (1:500, Alexa Fluor^®^ 488 Donkey Anti-Goat, ab150129, Abcam, United States), and activated neurons were labeled with anti-cfos (#2250S, Cell Signaling Technology, United States) + red fluorescent secondary antibody (1:500, SA00013-8, CoraLite594 Donkey Anti-Rabbit, Proteintech, China). In HM3D and HM4D groups, GLP-1 neurons were labeled with anti-DsRed (632496, TAKARA, Japan) + red fluorescent secondary antibody (1:800, SA00013-4, CoraLite594 Goat Anti-Rabbit, Proteintech, China) and activated neurons were labeled with anti-cfos (1:2000, ab208942, Abcam, United States) + green fluorescent secondary antibody (1:200, SA00013-1, CoraLite488 Goat Anti-Mouse, Proteintech, China). The co-expression of GLP-1 neurons and cfos in the neuron cells indicated that GLP-1 neurons were activated. The activated GLP-1 neurons in the NTS area of each group were counted and statistically analyzed. The protein expression of differential genes in the hypothalamus of rats in each group was demonstrated by immunofluorescence single labeling. The tissue of the hypothalamus was prepared into frozen slices with a thickness of 20 μm, cubed with anti-Bgn (1:100, 16409-1-AP, Proteintech, China) or anti-Mapkapk3 (1:200, 15424-1-AP, Proteintech, China), and labeled with a green fluorescent (1:500, SA00013-2, CoraLite488 Goat Anti-Rabbit, Proteintech, China). Nuclei were stained with DAPI and observed under a fluorescence microscope. The expression of Bgn and Mapkapk3 in the hypothalamus was analyzed by the imagepro plus 6.0 software with integrated optical density (IOD) for fluorescence intensity analysis.

### Statistical analysis

SPSS 25.0 software package was used for statistical analysis. The results were expressed as mean ± standard deviation (x ± s). One-way analysis of variance was used for comparison between groups within the same time period. If there was an overall difference, multiple comparisons with the Tukey’s method were used. Repeated measures analysis of variance was used to compare different time points within the same group, and if there were differences, multiple comparisons with the Tukey’s method were used. *P* < 0.05 was considered significant.

## Results

### Differentially expressed gene analysis results

**FIGURE 2:**
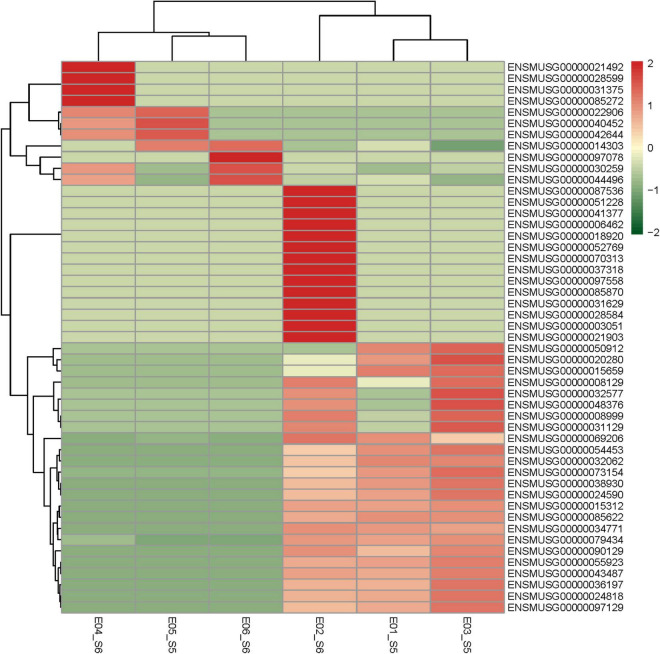
Heatmap of DEGs. Red areas represent highly expressed genes and green areas represent lowly expressed genes. DEGs, differentially expressed genes.

Based on the screening criteria of *P* < 0.05, |logFC |> 1, 49 related differentially expressed genes were analyzed in the GSE135862 dataset. Among them, there were 11 upregulated differentially expressed genes and 38 downregulated differentially expressed genes. According to the expression fold of the differential genes, the related genes with the largest differential fold were selected, which then upregulated *Bgn* and down-regulated *Mapkapk3*, respectively. A specific heatmap can be seen in Figure 2.

### Gene ontology and Kyoto encyclopedia of genes and genomes pathway enrichment analysis results of differentially expressed genes

**FIGURE 3:**
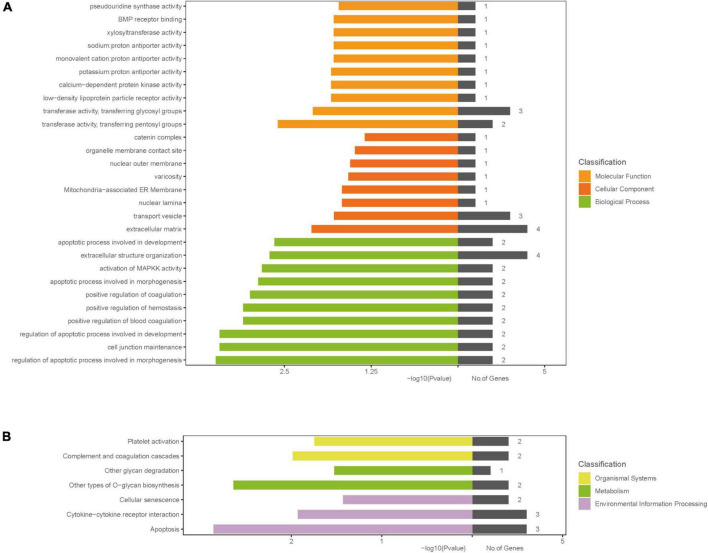
Gene ontology and KEGG enrichment analysis. **(A)** GO enrichment result of DEGs; **(B)** the results of KEGG signaling pathway enrichment analysis; The *x*-axis label represents *P*-value and the number of genes and the *y*-axis label represents GO and KEGG terms, different colors represent different classification. DEGs, differentially expressed genes; GO, gene ontology; KEGG, Kyoto Encyclopedia of Genes and Genomes pathway.

The results of GO analysis showed that the molecular functions of differential genes mainly included pseudouridine synthase activity, BMP receptor binding, xylosyltransferase activity, proton antiporter activity (sodium, monovalent cation, potassium), calcium-dependent protein kinase activity, low-density lipoprotein particle receptor activity, transferase activity, and transferring glycosyl and pentosyl groups; differential genes were mainly localized in seven regions around the cells, which were catenin complex, organelle membrane contact site, nuclear outer membrane, varicosity, mitochondria-associated ER membrane, nuclear lamina, and transport vesicle and extracellular matrix; and the biological pathways in which differential genes were involved included apoptotic process involved in development, extracellular structure organization, activation of MAPKK activity, apoptotic process involved in morphogenesis, positive regulation of coagulation, positive regulation of hemostasis, positive regulation of blood coagulation, regulation of apoptotic process involved in development, cell junction maintenance, and regulation of apoptotic process involved in morphogenesis. The results of KEGG signaling pathway enrichment analysis showed that differential genes played an important role in platelet activation pathway, complement and coagulation cascades pathway, other glycan degradation pathway, other type of O-glycan biosynthesis pathway, cellular senescence pathway, cytokine-cytokine receptor interaction pathway, and apoptosis pathway. Specific results can be seen in Figure 3.

### Protein-protein interaction networks and important genes of differentially expressed genes

**FIGURE 4:**
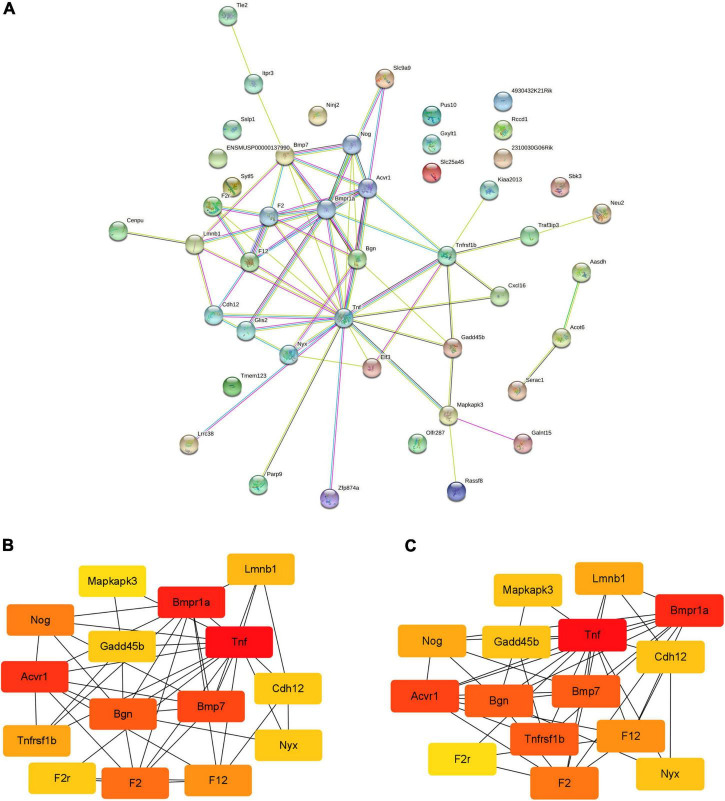
The PPI networks of DEGs and modules. **(A)** PPI network analyzed by STRING database; **(B,C)** the top 15 genes selected according to the MCC and degree method. PPI, protein-protein interaction; DEGs, differentially expressed genes; MCC, maximal clique centrality.

The PPI network of DEGs and its modules are shown in Figure 4. With the STRING database, 46 of 49 DEGs were mapped onto a PPI network. There are 46 nodes and 66 edges (Figure 4A). After the PPI network is imported into Cytoscape, the key nodes and subnetworks in the PPI network are predicted and explored by several topological algorithms using the cytoHubba plugin. The top 15 genes selected according to the MCC and degree method were *F12*, *Gadd45b*, *Cdh12*, *Nyx*, *Mapkapk3*, *Bgn*, *Tnf*, *Lmnb1*, *Tnfrsf1b*, *F2r*, *Bmpr1a*, *Acvr1*, *Bmp7*, *Nog*, and *F2* (Figures 4B,C).

### Different glucagon-like peptide 1 neuronal excitability modulates appetite-related behavior

**FIGURE 5:**
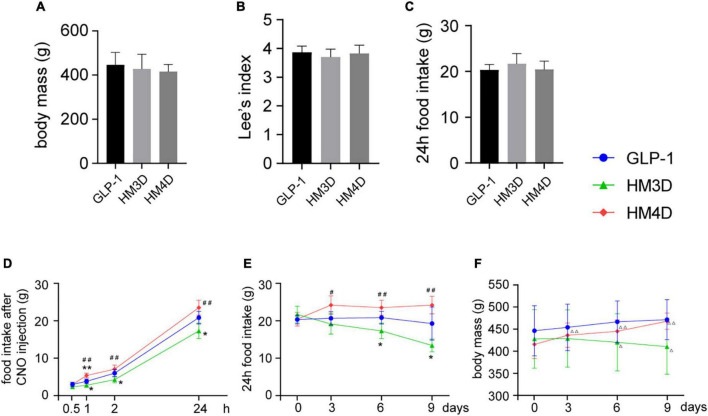
Appetite-related behavioral changes in each group. **(A–C)** Baseline of body mass, lee’s index, and 24 h food intake of rats in each group, which were collected at day 0; **(D)** cumulative food intake of rats in each group at different time points after the first CNO injection; **(E)** changes in 24 h food intake of rats in each group after CNO injection during the intervention period; **(F)** changes in body mass of rats in each group during the intervention period; vs. GLP-1 group, *P < 0.05, **P < 0.01; vs. HM3D group, ^#^*P* < 0.05, ^##^*P* < 0.01; vs. day 0, ^△^ *P* < 0.05, ^△^^△^ *P* < 0.01. CNO, Clozapine-N-oxide.

As shown in Figures 5A–C, there was no significant difference in body weight, Lee’s index, and 24-h food intake among the three groups before intervention (*P* > 0.05). After the first time of CNO injection, the feeding behavior of the three groups of rats showed significant differences (Figure 5D). Compared with the GLP-1 group, rats in the HM3D group showed a significant reduction in food intake from 1 h after CNO injection (*P* < 0.05), and the cumulative food intake was significantly lower than that in the GLP-1 group at 1 h, 2 h, and 24 h after CNO injection (*P* < 0.05 or *P* < 0.01). Compared with the GLP-1 group, the HM4D group showed a significant increase in food intake only at 1 h after CNO injection (*P* < 0.01); compared with the HM3D group, the food intake of the HM4D group increased significantly at 1, 2, and 24 h after injection (*P* < 0.05 or *P* < 0.01). There was no statistical difference in the 24-h food intake before CNO injection among the groups of rats, which showed the highest food intake in the HM4D group and the lowest food intake in the HM3D group on days 3, 6, and 9 of injection (Figure 5E). The body weights of rats in the three groups showed different trends during the experiment. Rats in the GLP-1 group did not show significant variation in body weights after CNO injection. The body weights of rats in the HM3D group were lower than the baseline since the 6th day after CNO injection (*P* < 0.05), and the body weights of rats in the HM4D group increased since the 3rd day after CNO injection. However, we did not observe significant differences in body weight among the three groups of rats at each time point (Figure 5F).

### Chemical genetics technology can activate or inhibit glucagon-like peptide 1 neurons in the nucleus tractus solitarius

**FIGURE 6:**
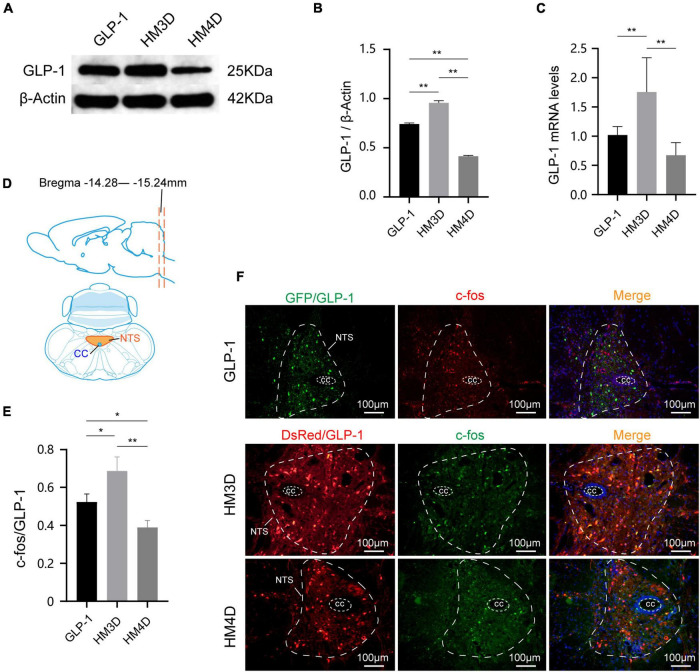
Comparison of protein and gene expression of GLP-1 in NTS. **(A,B)** WB and protein expression of GLP-1in NTS; **(C)** gene expression of GLP-1 in NTS; **(D)** the slice range and localization pattern of the coronal plane where the NTS is located; **(E)** comparison of activation numbers of GLP-1 neurons in NTS; **(F)** representative figures of activated GLP-1 neurons in the NTS of rats in each group; *P < 0.05, **P < 0.01. WB, Western blotting; GLP-1, glucagon-like peptide 1; cc, central canal; NTS, nucleus of the solitary tract.

As shown in Figures 6A,B,C, both the protein and gene expression of GLP-1 in NTS were upregulated in the HM3D group, while in the HM4D group were downregulated. As shown in Figures 6D,F, the GLP-1 neurons in the NTS region of the three groups of rats were successfully labeled with GFP or DsRed primary antibody, and the GLP-1 neurons expressed different amounts of c-fos. The number of activated GLP-1 neurons in the HM3D group was significantly higher than that in the GLP-1 group and the HM4D group (*P* < 0.05); the number of activated neurons in the GLP-1 group was higher than that in the HM4D group (*P* < 0.05) (Figure 6E). The results indicated that CNO injection could effectively activate or inhibit GLP-1 neurons in NTS.

### Different excitatory properties of glucagon-like peptide 1 neurons regulate the protein and gene expression of biglycan and mitogen-activated protein kinase activated protein kinase 3 in the hypothalamus

**FIGURE 7:**
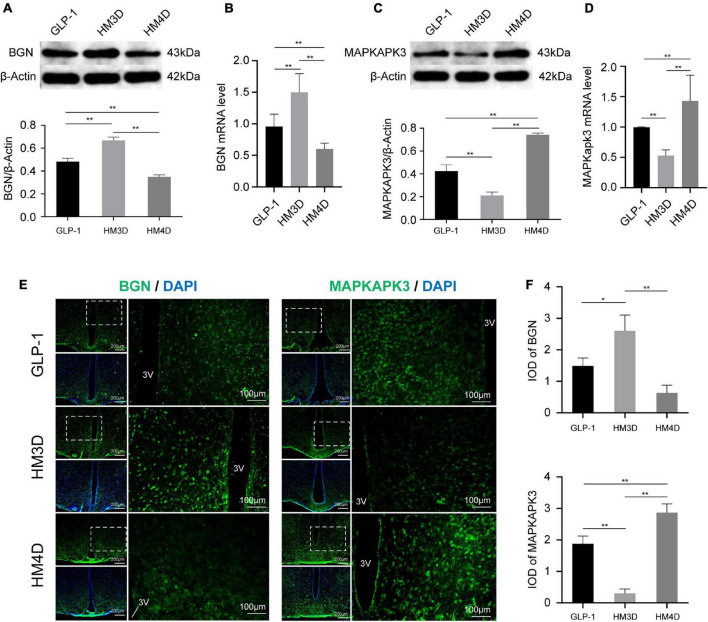
Comparison of protein and gene expression of Bgn and Mapkapk3 in the hypothalamus. **(A)** WB and protein expression of Bgn in hypothalamus; **(B)** gene expression of Bgn in hypothalamus; **(C)** WB and protein expression of Mapkapk3 in hypothalamus; **(D)** gene expression of Mapkapk3 in hypothalamus; **(E)** representative figures of Bgn and Mapkapk3 in hypothalamus of rats in each group; **(F)** comparison of IOD of Bgn and Mapkapk3 in hypothalamus; *P < 0.05, **P < 0.01. 3V, the 3rd ventricle. WB, Western blotting; Bgn, biglycan; Mapkapk3, mitogen-activated protein kinase activated protein kinase 3; IOD, integrated optical density.

As shown in Figures 7A,B, the activation of GLP-1 neurons upregulated the gene and protein expression of Bgn in the hypothalamus (*P* < 0.01); while suppressing it induced the opposite effect (*P* < 0.01). On the contrary, activated GLP-1 neurons can downregulate the gene and protein expression of Mapkapk3 in the hypothalamus (*P* < 0.01), while suppressing GLP-1 can activate Mapkapk3 (*P* < 0.01) (Figures 7C,D). Observing the fluorescence images of the hypothalamus, it was found that both Bgn and Mapkapk3 proteins were widely expressed in the hypothalamus without obvious cell specificity (Figure 7E). By analyzing the fluorescence intensity, we found that the expression of Bgn increased after activation of GLP-1, while Mapkapk3 decreased (Figure 7F), which was consistent with the results of WB and qPCR.

## Discussion

Glucagon-like peptide 1, a peptide hormone from the intestinal tract, plays a central role in the coordination of postprandial glucose homeostasis (Gribble and Reimann, 2021). Intake and digestion of carbohydrates, fats, proteins, and bile acids are the main physiological stimuli that promote GLP-1 secretion (Gribble and Reimann, 2019). GLP-1 biosynthesis and release are performed by enteroendocrine cells of the intestine, L cells (Song et al., 2019). L cells are more distributed in the jejunum, ileum, and colon (Sjolund et al., 1983). Therefore, the stimulation of L cells occurs downstream of food digestion, which is closely related to the local rate of nutrient absorption of the intestine and can control gastric emptying and intestinal tract creeping through feedback regulation (Gribble and Reimann, 2021). Therefore, its main functions include not only stimulating insulin secretion and inhibiting glucagon secretion but also regulating gastrointestinal motility, and working together in many ways to maintain blood glucose homeostasis. In addition to being secreted by the intestine, GLP-1 also exists in neurons of NTS, which release GLP-1 in the central nervous system, including the project to the feeding centers in the hypothalamus (Llewellyn-Smith et al., 2011). GLP-1-mediated neural circuits are the core mechanism by which GLP-1 neurons in NTS exert their appetite-suppressing effects (Cheng et al., 2020). Therefore, it is also a physiological regulator of appetite and food intake (Holst, 2007).

Glucagon-like peptide 1 exerts its physiological function by binding to a dedicated G-protein-coupled receptor, GLP-1R, expressed in a variety of cell types (Thorens, 1992). Numerous attempts have been made to identify alternative GLP-1R or subtypes, but at present only a single GLP-1R has been identified, whether expressed in the brain, the stomach, or the pancreas (Wei and Mojsov, 1995). In addition to being expressed in the gastrointestinal tract, pancreas, and brain, studies have shown that GLP-1R can be also detected in the vagal afferent nerves, scattered atrial cardiomyocytes, vascular smooth muscle, lung, and some immune cells (Pyke et al., 2014; Richards et al., 2014; He et al., 2019). The lateral hypothalamic GLP-1R has been identified as an indispensable element of normal food reinforcement, food intake, and body weight regulation (Lopez-Ferreras et al., 2018). As mentioned earlier, GLP-1R-expressing vagal afferent cells have been found to form fibers within the intestinal villi adjacent to enteroendocrine cells. Therefore, it is generally believed that GLP-1R is a chemosensory neuron of the vagal afferents nerve, which helps the vagal afferents nerve receive nutrient detection information in a paracrine manner by binding to GLP-1 released locally by enteroendocrine cells (Krieger et al., 2016; Grill, 2020). Meanwhile, the activity of GLP-1R in vagal afferents also encodes the feeling of distention in the gastrointestinal tract through the inner ganglia of myenteric plexus intramuscular array detection in gastric and intestinal smooth muscle layers (Williams et al., 2016; Bai et al., 2019). Thus, the vagal afferents of the intestine receive gastrointestinal stimulation through both chemoreceptors and mechanoreceptors to generate neural signals toward the corresponding nuclei of the brainstem, such as those in the NTS and posterior area (AP), then to the hypothalamus, which releases GLP-1 and activates the receptor. However, the current link of this endogenous peripheral GLP-1 indirectly interacting with the central GLP-1 system (*via* the vagal and/or endocrine pathways) remains controversial, and the necessary intestine-brain circuit has not been empirically confirmed (Brierley et al., 2021). Therefore, further research in this field should be conducted in the future.

Glucagon-like peptide 1 forms the basis for a variety of current drugs for the treatment of type 2 diabetes and obesity, as well as new agents currently being developed. GLP1-RAs are used clinically to treat T2DM and promote weight loss in people with obesity (Knudsen and Lau, 2019). Although the identity of the GLP-1R-expressing target cells underlying appetite suppression remains under discussion, powerful evidence indicates that GLP1-RAs exert their effects by targeting GLP-1R in the brainstem and/or hypothalamus (Knudsen and Lau, 2019). The mechanism of action of GLP-1 and its receptor in the brain is still unclear, which has caused some difficulties in the optimization of drug development and obesity treatment. Therefore, this study aimed to investigate the differential genes in the brain after GLP1-RAs treatment. After bioinformatics analysis, we found two significantly different genes, Bgn of upregulated genes and Mapkapk3 of downregulated genes.

Biglycan, a class I small leucine-rich proteoglycans, is a key component of the extracellular matrix (ECM) that participates in scaffolding the collagen fibrils and mediates cell signaling. Dysregulation of Bgn expression can result in a wide range of clinical conditions such as metabolic disorder, inflammatory disorder, musculoskeletal defects, and malignancies (Appunni et al., 2021). ECM disorganization is a pivotal step in metabolic disorders like obesity which involves accommodating the expanding adipose tissue mass (Kim et al., 2016). The ECM in these states releases non-fibrillar proteoglycans such as Bgn, which take part in receptor-mediated interactions to regulate inflammation and organ-specific metabolic disturbances. The ECM plays a role in regulating neurite outgrowth, and neural function and plasticity as well as metabolic diseases (Dityatev et al., 2010). Also, ECM remodeling is crucial for regulating the morphogenesis of the intestine (Bonnans et al., 2014). In this study, GO analysis also found differential genes enriched in ECM. KEGG analysis showed that differential genes play an important role in other types of glycans degradation and biosynthesis pathway. So, the upregulation of Bgn expression in this study may indicate increased ECM remodeling, and as previously mentioned, changes in intestinal mechanical signaling can regulate gastrointestinal motility and food intake.

Mitogen-activated protein kinase activated protein kinase 3 is a member of the Mapk signaling pathway family (Wei et al., 2015). The Mapk family is significant in various cellular processes such as apoptosis, oxidative stress, differentiation, inflammatory response, and proliferation, and includes three members: extracellular signal-regulated kinase (Erk1/2), p38, and c-Jun N-terminal kinase (JNK) (Miloso et al., 2008). p38 Mapk is the best-characterized kinase upstream of Mapkapk3 (Freshney et al., 1994). Studies have shown that p38Mapk can promote β-cell exhaustion in the pancreas, and β-cells can express GLP-1R (Manieri and Sabio, 2015). At the same time, p38 can also regulate the secretion of insulin, and the insulin level in p38-deficient mice is increased. Then, in this study, Mapkapk3 is a downstream factor of p38, and the downregulation of Mapkapk3 may inhibit the p38 Mapk pathway, thereby promoting insulin secretion and increasing the expression of GLP-1R. Studies have shown that the silencing of *Bgn* gene can upregulate p38Mapk (Appunni et al., 2021). In the verification of bioinformatics analysis results, we found that the expression of Bgn mRNA was upregulated and the expression of Mapkapk3 mRNA was downregulated when the GLP-1 neurons were activated, which implied the inhibitory effect of BGN in regulating the p38 pathway from another perspective.

## Conclusion

In summary, we screened out the key differential genes that affect feeding behavior after GLP-1R activation through bioinformatics analysis. Bgn and Mapkapk3 were found to be more associated with changes in feeding behavior. Changes in Bgn and Mapkapk3 in the hypothalamus were also detected after activating or inhibiting GLP-1 neurons in the NTS by chemogenetic treatment, which were consistent with the results of bioinformatics analysis. It shows that Bgn and Mapkapk3 may be important downstream cytokines of GLP-1 in regulating appetite, which were worthy of further research in the future.

## Data availability statement

The raw data supporting the conclusions of this article will be made available by the authors, without undue reservation.

## Ethics statement

The animal study was reviewed and approved by the Institutional Animal Care and Use Committee of Wuhan University.

## Author contributions

QS, JT, and YY designed the study protocol and obtained the funding. YS and YH were responsible for the bioinformatics analysis part, animal experimentation, and basic experiment parts. QS was responsible for data analysis. QS, JT, and YS drafted this manuscript. All authors have read and approved the final manuscript, adhere to the trial authorship guidelines, and agreed to publication.

## Footnotes

1
https://david.ncifcrf.gov/

2
https://cn.string-db.org/

## Abbreviations

GLP-1: glucagon-like peptide 1
GEO: gene expression omnibus
GO: gene ontology
KEGG: Kyoto encyclopedia of genes and genomes pathway
PPI: protein-protein interaction
DEGs: differentially expressed genes
NTS: nucleus tractus solitarius
Bgn: biglycan
Mapkapk3: mitogen-activated protein kinase activated protein kinase 3
TCM: traditional Chinese medicine
CCK: cholecystokinin
GLP1-RA: GLP-1 receptor agonist
RIP-Seq: RNA co-immunoprecipitation combined with high-throughput sequencing
DVC: dorsal vagal complex
MBH: medial basal hypothalamus
logFC: log value of gene fold change
BP: biological process
MF: molecular function
CC: cellular component
FDR: false discovery rate
MCC: maximal clique centrality
rAAV: adeno-associated virus
cre: cyclization recombination enzyme
CNO: clozapine-N-oxide
WB: Western blotting
RT-qPCR: reverse transcription-quantitative polymerase chain reaction
cDNA: complementary DNA
IF: immunofluorescence
IOD: integrated optical density
AP: posterior area
ECM: extracellular matrix
Erk1/2: extracellular signal-regulated kinase
JNK: c-JunN-terminal kinase.
